# Comparison Study on the Adsorption Capacity of Rhodamine B, Congo Red, and Orange II on Fe-MOFs

**DOI:** 10.3390/nano8040248

**Published:** 2018-04-17

**Authors:** Fuhua Wei, Ding Chen, Zhao Liang, Shuaiqi Zhao

**Affiliations:** 1 College of Materials Science and Engineering, Hunan University, Changsha 410082, China; wfh.1981@163.com (F.W.); walleliang@163.com (Z.L.); 15574824279@163.com (S.Z.); 2 College of Chemistry and Chemical Engineering, Anshun University, Anshun 561000, China

**Keywords:** wastewater treatment, metal-organic frameworks, dye removal, microwave-assisted ball milling

## Abstract

Using a microwave-assisted ball-milling approach, Fe-based metal-organic frameworks (Fe-MOFs) were prepared from FeSO_4_·7H_2_O and trimesic acid. Scanning electron microscopy, Fourier-transform infrared spectrometry, X-ray, and thermogravimetric analysis were utilized to characterize the thermal stability and structure of the prepared Fe-MOFs. These Fe-MOFs were used to remove organic dyes from aqueous solutions. Specifically, they removed 96.97% of 23.3592 mg/L of Congo red in a 200 mL solution within 300 min of treatment with natural light at 15 °C. Likewise, 88.21 and 70.90% of 22.7527 mg/L of Orange II and 17.8326 mg/L of Rhodamine B, respectively, were removed from 200 mL solutions within 300 min of treatment at 15 °C. At 35 °C, 99.57, 95.98, and 99.38% of 23.3855 mg/L of Congo Red, 22.7365 mg/L of Orange II, and 17.9973 mg/L of Rhodamine B, respectively, were removed from 200 mL solutions within 300 min of treatment. The adsorption kinetics were investigated and the pseudo-first-order kinetic model was found to be superior to the pseudo-second-order kinetic model. Overall, using metal-organic frameworks to treat dye wastewater was found to be inexpensive, feasible, and efficient. Therefore, this material has future prospects in research and applications in the purification of wastewater.

## 1. Introduction

Clean water is a critical societal provision. The environment is currently significantly threatened by pollution, which is increasing daily as a result of continuing economic development, rapid urbanization, and a steadily increasing world population [1]. There is also a continuously increasing wasting of water resources made worse by water contamination with various pollutants, including inorganic and organic chemicals. Hexavalent chromium [2], dyes [3], herbicides/pesticides [4], and aromatics/organics [5,6] are typical chemicals that must be removed from wastewater. Of the dyes, many are toxic and even carcinogenic [7,8]. Rhodamine B (RhB), Orange II, and Congo red (CR) are common examples of organic dyes found in wastewater and industrial effluents. These dyes are widely used in rubbers, carpets, textiles, cosmetics, paper, plastics, and food. Due to their toxicity, these dyes directly destroy microbes or at least inhibit microbial catalytic performance.

In order to remove dyes from wastewater, a number of biological, chemical, catalysis [9,10,11,12,13,14,15], and physical methods have been investigated [7]. Common adsorbent materials include activated carbon, activated alumina, molecular sieves, metal hydroxides, silica gel, and metal-organic frameworks (MOFs). MOFs, which consist of metal-oxo clusters or metal ions and organic linkers, have fascinating crystalline structures, tailorable chemistry, large specific surface areas, and good porosity [16]. MOFs have attracted much attention due to their uses in adsorption and catalysis and potential to be designed with a specific pore shape and size from multifunctional ligands and metal ions or metal ion clusters. The performances of MOFs can be controlled, to some extent, by cautiously tuning their structures and functionalities to allow for distant targets to be reached or to improve adsorbent properties. Another attractive characteristic of MOFs is the tunability of their physiochemical performance after the involved crystalline materials have already been formed [17,18,19,20].

Since the 1990s, MOFs have garnered significant research interest, not only due to their unique chemical structures and characteristics, but also their potential applications in a variety of research fields, including polymerization [21], drug delivery/biomedicine [22], catalysis [23], luminescence [24], adsorption/storage in the gas phase [25,26,27], chemical separation [16,28,29], magnetism [30], and liquid phases [31]. Recently, MOFs have also been recognized as prospective materials for the liquid-phase adsorption of various hazardous compounds [32], including sulfur- [33,34] and nitrogen- containing compounds [35], in both aqueous and non-aqueous media. MOFs can be prepared using microwave [36], chemical mechanical polishing [37], hydrothermal [38], ultrasonic [39], and mechanochemical methods [40] among others.

Microwave-assisted ball milling was first described by our laboratory and is based on a solid-liquid ball-milling (MSBM) approach that involves using a ball-milling machine in a microwave oven [41,42,43,44,45,46]. The coupling of mechanical milling with microwaves enables certain nanocrystal materials, such as magnetic and metal oxidation ferrite, to be generated at room temperature [47,48,49]. In the present study, MOF-based adsorptive removal of hazardous compounds, including RhB, Orange II, and CR, from aqueous media was investigated.

## 2. Experimental

### 2.1. Synthesis and Characterization of Experimental Materials

Fe-MOFs were synthesized by microwave-assisted ball milling on the basis of a previously reported way [48]. H_3_BTC (98%) and FeSO_4_·7H_2_O were purchased from Shanghai Aladdin Biological Technology Co., Ltd. (Shanghai, China), while potassium dichromate (≥99.8%) was purchased from Tianjin Zhiyuan Chemical Reagent Co., Ltd. (Tianjin, China). FeSO_4_·7H_2_O (0.0537 mol, 14.9297 g), trimesic acid (0.0358 mol, 7.5187 g), stainless steel balls (1350 g), and 800 mL deionized water were combined in a tetrafluoroethylene milling pot. Stir milling at 200 rpm and the microwave oven were started concurrently. After 40 min, the solution converted from a colorless liquid into a white solid and was stable in this form. This solid white compound was filtered, washed with water, added to a beaker containing ethanol, and then stirred with a magnetic stirrer for 3 h. This mixture was then filtered by suction and dried, and then the final product was collected, characterized, and used in experimental reactions.

Structure and morphology were characterized using X-ray diffraction (D-5000, Siemens, Chicago, IL, USA, Cu-Kα radiation), Fourier-transform infrared spectroscopy (IRTracer-100, SHIMADZU, Shanghai, China), and field emission scanning electron microscopy (JSM-6700F, Tokyo, Japan). The particle thermogravimetric curves were obtained in an argon atmosphere at temperatures from 35 to 700 °C increasing at a rate of 5 °C/min using a NETZSCH STA 449C thermal analyzer (Selb, Germany).

### 2.2. Removal of Organic Dyes

Organic dyes were removed at approximately 15 °C and 35 °C in a 500 mL beaker. Sample (200 mg) was mixed with 200 mL of an approximately 20 mg/L organic dye aqueous solution while exposed to natural light and magnetic stirring. Every 30 min, a 10-mL sample of the dye solution was assessed at 220 V by ultraviolet spectrometry using a UV-2550 from Shimadzu Instruments Co., Ltd. (Suzhou, China). The rate of dye removal was determined using C = (C_0_ − C_t_)/C_0_ × 100%, where C_0_ represents the initial concentration of dye and C_t_ represents the concentration of dye after t minutes.

RhB, CR, and Orange II concentrations were measured using the UV-Vis spectrophotometer (UV-2550) at wavelengths of 554, 495.5, and 484.5 nm. The amount of adsorbed organic dye was calculated using the following equation [50].

(1) $$q_{e}=\frac{C_{0}-C_{e}}{m}v$$

where C_0_ and C_e_ are the initial and equilibrium concentrations of Cr(VI) in solution (ppm), respectively, V is the solution volume (L), and m is the adsorbent mass (g).

## 3. Results and Discussion

### 3.1. Synthesis and Characterization of Fe-MOFs

In the present study, the H_3_BTC and FeSO_4_·7H_2_O reacted over the course of a few minutes during MSBM. After 40 min, the color of the reaction remained unchanged, indicating that Fe-MOFs production was complete. Notably, MSBM incurred faster reaction rates than observed in conventional synthesis approaches of generating Fe-MOFs [48,51], such as the solvothermal approach, where the rate was similar to other microwave-assisted approaches [52].

The reaction mechanism was simple. Due to the coupling of ball milling and microwaves, the H_3_BTC anions formed quickly through ionization in aqueous solutions and then attacked the metal cations from the salt, thus forming a coordination compound [53], primarily due to the carboxylate forming extended conjugate bonds that render the two oxygen atoms equivalent. However, the density of the electron cloud should have a symmetrical distribution. Because the three carboxyls groups are connected to the benzene ring when protonated, the resulting extended-bonds could more easily coordinate with metal ions and form coordination polymers.

**Figure 1:**
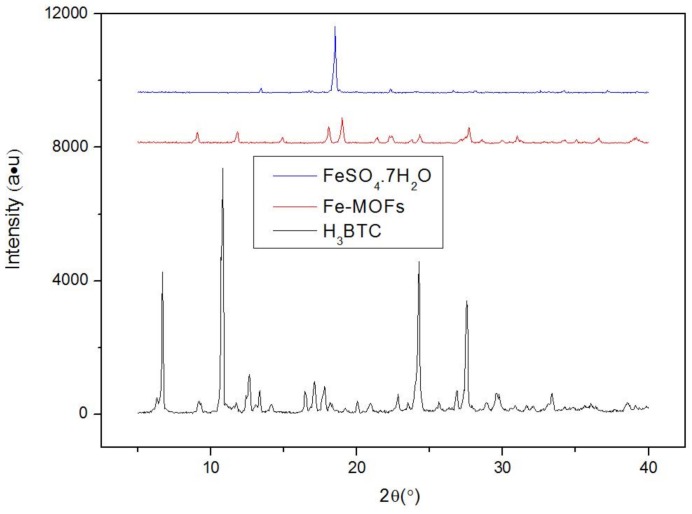
XRD of Fe-based metal-organic frameworks (Fe-MOFs).

**Figure 2:**
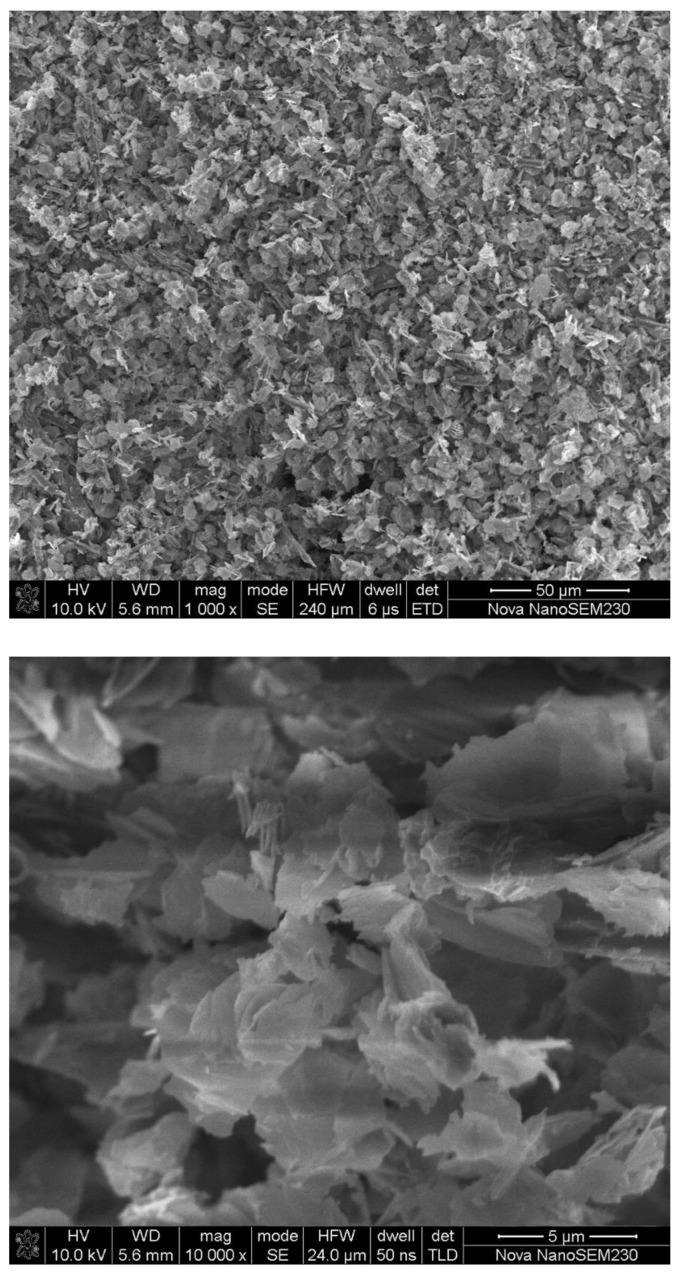
SEM images of Fe-MOFs.

The X-ray diffraction Fe-MOF spectrum presented in Figure 1 contains twelve strong absorption peaks. The three strong peaks present in the post-reaction spectrum did not overlap with those in the pre-reaction spectrum, confirming the formation of new materials. The morphology of the Fe-MOFs was assessed by scanning electron microscopy and is shown in Figure 2. At 1000×, randomly packed blade-shaped particles were observed. At 10,000×, the morphologies of the individual particles could be visualized.

**Figure 3:**
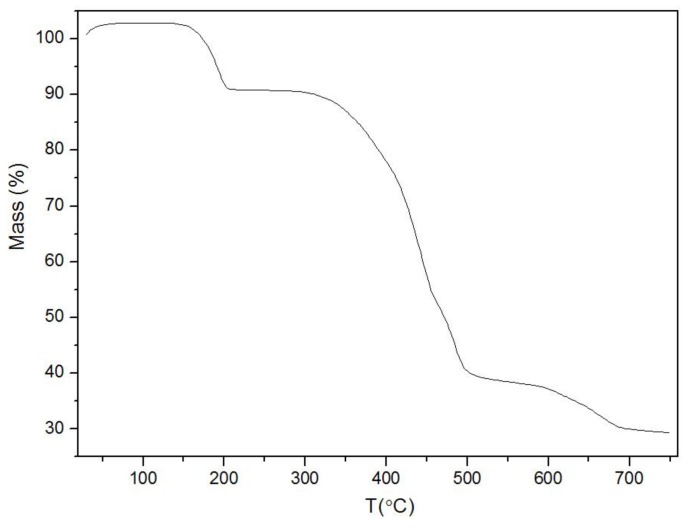
Thermal analysis of Fe-MOFs.

Thermogravimetric analysis was carried out using a quartz pan. As shown in Figure 3, weight loss occurring in the argon atmosphere could be divided into two separate stages. The first stage, which occurred before 167 °C, was due to the loss of residual solvent molecules from the framework material from the solution-based synthesis [54,55]. The second stage, which started at 392 °C, occurred when the chemical bonds began to break and the carboxyl and benzene ring were lost. At 492 °C, the frame structure had completely collapsed.

**Figure 4:**
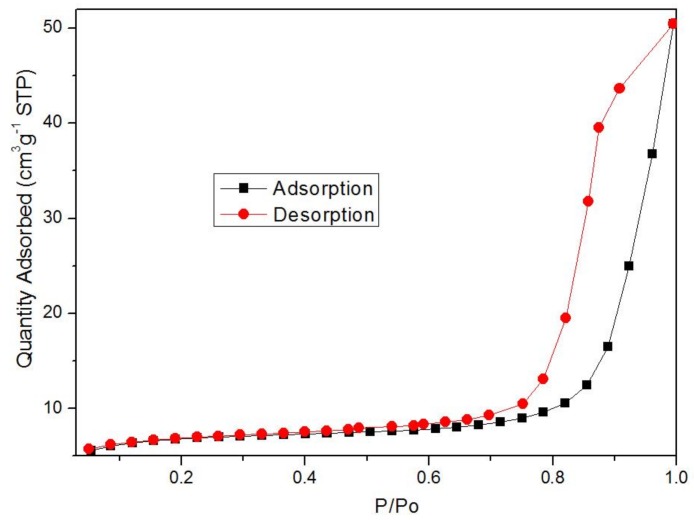
N_2_ adsorption–desorption isotherms of Fe-MOFs.

The N_2_ adsorption–desorption isotherms are shown in Figure 4. The specific surface areas was found to be 21.8900 m^2^/g, and the average pore diameter was 24.4233 nm. Moreover, it can be seen from Figure 4 that there are clear hysteresis loops indicating a mesoporous material. It can be seen from the figure that, before P/P_0_ = 0.7, N_2_ molecules were adsorbed on the inner surface of the mesoporous materials by a single layer to multiple layers. In the case of P/P_0_ = 0.8, the adsorption process increases, which reflects the size of the sample aperture and can also be used as the basis for the homogeneity of the mesoporous materials.

**Figure 5:**
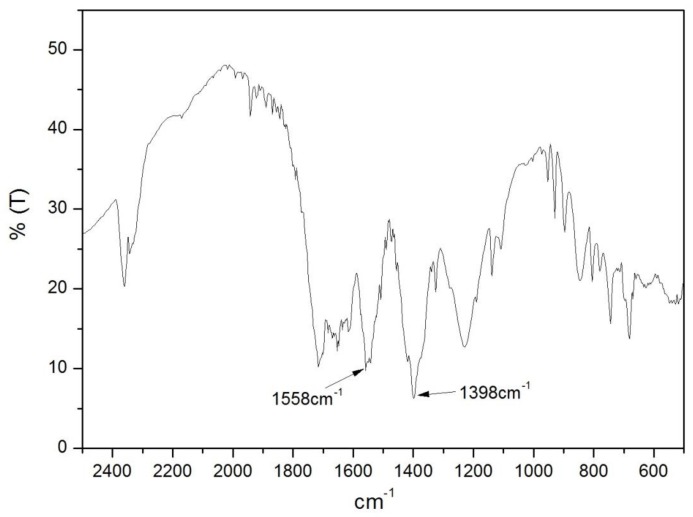
IR spectra of Fe-MOFs.

The structure of the Fe-MOF was tested via IR spectroscopy (as shown Figure 5). The corresponding peak of symmetric and anti-symmetric stretching vibrations to the carboxylate was observed at 1398 and 1558 cm^−1^, respectively. However, the electron density should be distributed symmetrically. When the three carboxyl groups in H_3_BTC are protonated because they take part in hydrogen bonding between inter- and intramolecular, the extended bonds of their corresponding anions can more easily form coordination polymers and metal ions.

### 3.2. Removal of Organic Dyes

**Figure 6:**
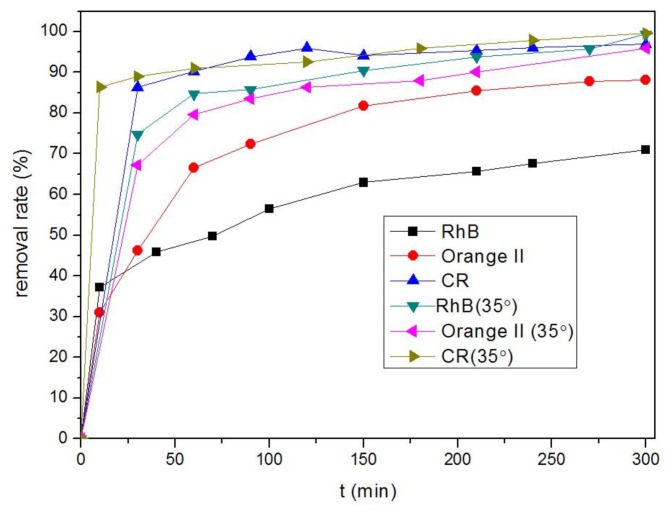
Removal rates of Rhodamine B (RhB), Orange II, and Congo red (CR) by Fe-MOFs.

Figure 6 shows that the removal efficiencies of RhB, Orange II, and CR by the MOFs reached 70.9, 88.21, and 96.97% at 15 °C and 99.38, 95.98, and 99.57% at 35 °C, respectively. Among these values, 86.28% of CR was removed within only 30 min. There are two major factors that influenced Fe-MOF adsorption of dyes. One factor is surface area, a factor that does not uniquely determine the adsorption capability of adsorbents. A second factor involved in the high adsorption capacity of Orange II and CR by Fe-MOFs was the π-electron donor/acceptor interactions with the MOF surfaces. Orange II and CR have C=C double bonds and π electrons. These π electrons are capable of easily interacting with the π electrons of the MOF-surface benzene rings through π-π electron coupling. These two dyes are also both cationic and anionic and exist as charged ions in an aqueous solution. Therefore, electrostatic attraction aids in the adsorption of dyes by Fe(II)-MOFs.

**Figure 7:**
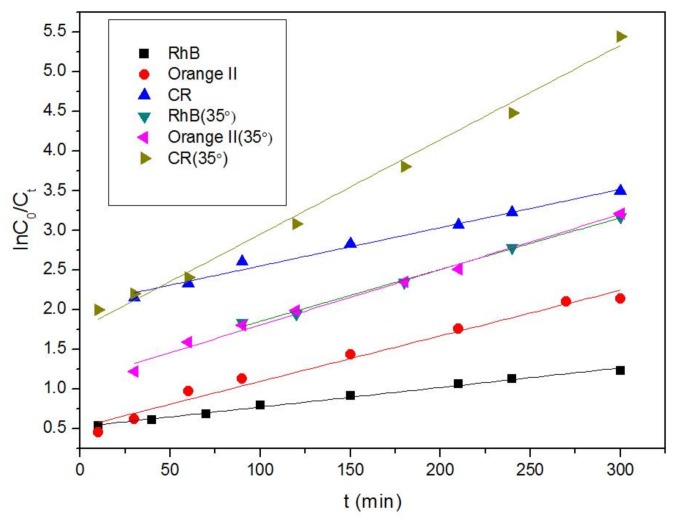
Pseudo-first-order plots of dye adsorption over Fe-MOFs.

The adsorption of RhB, Orange II, and CR by the Fe-MOFs was studied. Figure 7 presents the plots of the pseudo-first-order model, ln (C/C_0_) = kt, of the RhB, Orange II, and CR adsorptions by the Fe-MOFs at initial dye concentrations (C_0_) of 20 ppm. Table 1 presents the kinetic constants (k_1_) and correlation coefficients (R^2^) calculated. Pseudo-first-order kinetic constants (k_1_) for Orange II adsorption by Fe-MOFs were larger than those for the other dyes at 15 and 35 °C. However, the kinetic constant for RhB was smaller than that for the other dyes despite having a faster adsorption. The kinetic constants for Fe-MOFs indicate that rapid adsorption occurred in the presence of high concentrations of the dyes; a similar phenomenon has been reported in previous publications [56,57,58,59].

**Figure 8:**
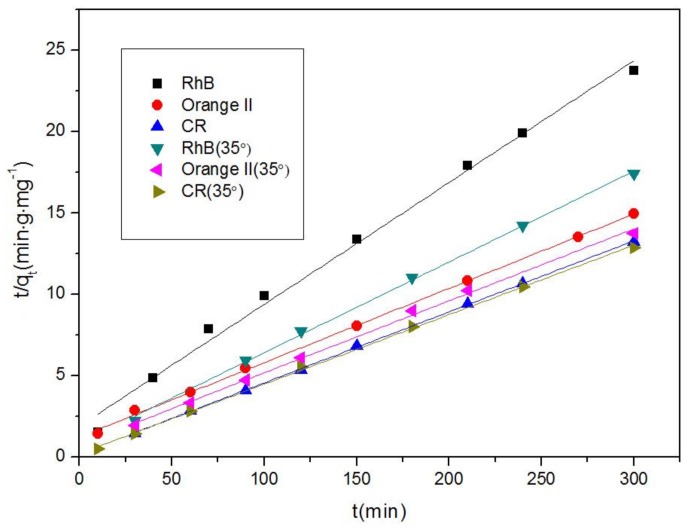
Pseudo-second-order plots of dye adsorption over Fe-MOFs.

Figure 8 presents the quantities (q_t_) of RhB, Orange II, and CR adsorbed by the Fe-MOFs over time (t), which were calculated using the following equations [50,60]:

(2) $$q_{t}=\frac{C_{0}-C_{t}}{m}v$$

(3) $$\frac{t}{q_{t}}=\frac{t}{q_{e}}+\frac{1}{k_{2}q_{e}^{2}}$$

where q_t_ and q_e_ represent the amounts (mg/g) of dye adsorbed by the adsorbents at time t and equilibrium, respectively. C_0_, C_t_, and C_e_ represent the concentrations of liquid-phase dye (mg/L) initially, at time t, and at equilibrium, respectively. Meanwhile, m (g) and V (L) are the quality of the adsorbents and the volume (L) of the dye solution, respectively.

As shown in Figure 7 and Figure 8, the adsorption capacity of the MOFs for the dye initially quickly increased, then gradually decelerated, and then reached adsorption equilibrium after prolonged contact. The kinetic constants (k_2_) and correlation coefficients (R^2^) calculated are presented in Table 1. There is the possibility that the original dye concentrations were high enough to serve as a driving force and surpass the resistance of the mass transfer between the liquidoid and solidoid [61].

The kinetics of adsorption is one of the most important parameters when describing an adsorbent. The results of this study indicate that adsorption of RhB, Orange II, and CR by Fe-MOFs is described well by the pseudo-first-order kinetic model and that the Fe-MOFs assessed are excellent at removing organic dyes. Dyes exist as charged ions in aqueous solutions and have benzene rings. Adsorption of dyes by Fe-MOFs may be a result of not only simple physical and chemical adsorption, but also the conjugation of dyes and MOFs. Dye contains C=C double bonds and π electrons and these π electrons can interact easily with the π electrons of Fe-MOF benzene rings through π-π electron coupling. Dye adsorption on the surface of the Fe-MOFs could occur with a face-to-face orientation through π-π conjugation until the equilibrium between adsorption and desorption has been reached. Therefore, electrostatic attraction also aids in dye adsorption by Fe-MOFs [62].

## 4. Conclusions

In summary, the microwave-assisted ball-milling approach proved to be an effective strategy and reliable for the synthesis of Fe-MOFs. The results show that the microwave-assisted ball milling process is an effective method for the simple and fast preparation of Fe-MOFs. The synthesised MOFs were measured for their capacity to remove organic dye solution from wastewater using natural light and the adsorption of dyes was analyzed with a pseudo-first-order model and a pseudo-second order model. By contrast, heating has good effect on the adsorption of dyes.

## Figures and Tables

**Table 1:** Kinetic constants (k) with correlation coefficients (R^2^).

Dyes		k_1_	k_2_	R^2^	
				Pseudo-First-Order Kinetic	Pseudo-Second-Order Kinetic
15 °C	RhB	0.00247	0.07488	0.99251	0.99248
	Orange II	0.00575	0.04568	0.97634	0.99899
	CR	0.00483	0.04368	0.98598	0.99979
35 °C	RhB	0.00653	0.05576	0.99513	0.99832
	Orange II	0.00698	0.04424	0.99231	0.99746
	CR	0.0119	0.04272	0.99242	0.99908
